# A comparative study between high ligation of the inter-sphincteric fistula tract via lateral Approach Versus Fistulotomy and primary sphincteroplasty in High Trans-Sphincteric Fistula-in-Ano: a randomized clinical trial

**DOI:** 10.1186/s12893-023-02117-0

**Published:** 2023-08-09

**Authors:** Philobater Bahgat Adly Awad, Basma Hussein Abdelaziz Hassan, Kerolos Bahgat Adly Awad, Beshoy Effat Elkomos, Mohamed Ali Mohamed Nada

**Affiliations:** General Surgery Department, Faculty of Medicine, Ain Shams University, Cairo, 2022 Egypt

**Keywords:** High trans-sphincteric fistula, Fistulotomy, Fistulectomy, Primary repair, Modified LIFT

## Abstract

**Background:**

Trans-sphincteric fistula management is very challenging and everyday new techniques are introduced to reach the safest and the most effective technique. In this study two of the most effective techniques are compared based on their post-operative outcomes.

**Objective:**

To compare the efficacy of high ligation of the inter-sphincteric fistula tract by lateral approach (modified LIFT) and Fistulotomy and primary sphincteroplasty (FIPS) in the management of high trans-sphincteric fistula regarding their post-operative outcomes in the form of post-operative pain, time of wound healing in weeks, wound infection, incontinence and recurrence within one year.

**Patients and methods:**

: The current study is single-blind, prospective, randomized, controlled, single-center trial conducted from June 2020 to June 2022 in the colorectal surgical unit of Ain Shams University Hospitals, which included 80 patients presented with high trans-sphincteric perianal fistula 55 (68.75%) males and 25 (31.25%) including a one-year follow-up postoperative.

**Results:**

There were 80 patients in our study 40 patients in each group. The mean age of group (I) is 46.65 with standard deviation 6.6. while, in group (II) the mean age is 45.85 with standard deviation 6.07 (*p* = 0.576). From the included 80 patients 55(68.7%) were males and 25 (31.25%) were females (*p* = 0.469). Regarding, postoperative wound infection occurred in 2(5%) Patients in group (I) and 7(17.5%) patients in group (II) (*p* = 0.154). There were no cases of incontinence in group I. However, there were 6(15%) cases of incontinence to gases only scored by Wexner score 3/20 in group II (*p* = 0.026) and its significant difference between the two techniques. Postoperative pain was assessed for one week duration by the visual analogue score (VAS) from 0 to 10 in which, zero is the least and 10 is the maximum. In group (I) 18(45%) patients scored their pain mild from 1 to 3, 20(50%) patients scored their pain moderate from 4 to 6 and 2(5%) patients scored severe pain from 7 to 9. While, in group (II) 14(35%) patients scored their pain mild from 1 to 3, 22(55%) patients their pain moderate from 4 to 6 and 4(10%) patients scored their pain severe from 7 to 9 (*p* = 0.275). Recurrence in one-year follow-up occurred in 13(32.5%) patients in group (I) about 7 patients had recurrence in the form of inter-sphincteric fistula and 6 patients in the form of trans-sphincteric fistula. While, in group II recurrence occurred in 1 (2.5%) patient in the form of subcutaneous fistula at the healing site (*p* = 0.001).

**Conclusion:**

Fistulotomy and primary sphincteroplasty is an effective and preferred technique for the trans-sphincteric fistula repair with high statistically significant lower incidence of recurrence in one-year follow-up as compared to modified LIFT technique. Although, there is higher incidence regarding incontinence to gases only post-operative. This work recommends fistulotomy and primary sphincter reconstruction procedure in high trans-sphincteric perianal fistulas to be more popular, to be implemented as a corner stone procedure along various and classic operations for such cases as it’s easy, feasible.

## Introduction

Anorectal fistulas are characterized by their tract location relative to the internal and external sphincters so a trans-sphincteric fistula is one that crosses to the other side of the external sphincter before exiting in the perianal area and thus involving both sphincters, Trans-sphincteric fistulas represent a challenge in management because of this and often require more complex or staged treatment [1].

High ligation of the inter-sphincteric fistula track by lateral approach is a new sphincter preserving technique for the treatment of anal fistula which was introduced by Chen et al. in 2012 to overcome disadvantages of the traditional LIFT technique [2, 3].

However, Conventional fistulotomy is a commonly used procedure and is still relied on by the majority of surgeons as the gold standard for the treatment of perianal fistula, so compared to other treatment modalities for complex trans-sphincteric anal fistula found in literature, it had been found that one stage surgery, which is Fistulotomy and primary sphincteroplasty has good results regarding healing of the fistula with acceptable risk of incontinence, relatively less recurrence rate and good wound healing [4, 5].

## Aim of work

The aim of the work is to compare the efficacy of high ligation of the inter-sphincteric fistula tract by lateral approach (modified LIFT) and Fistulotomy and primary sphincteroplasty in the management of high trans-sphincteric fistula regarding their post-operative outcomes in form of post-operative pain, wound healing in weeks, wound infection, incontinence and recurrence within one year follow-up.

## Patients and methods

The current study is single-blind, prospective, randomized, controlled, single-center trial conducted from June 2020 to June 2022 in the colorectal surgical unit of Ain Shams University Hospitals, which included 80 patients presented with high trans-sphincteric perianal fistula 55 (68.75%) males and 25 (31.25%) including a one-year follow-up postoperative.

### Randomization and blinding

Randomization was performed the day before surgery. Patients were randomized using a computer-generated randomization code and assigned either to experimental Group I for high ligation of the inter-sphincteric fistula tract by lateral approach procedure or experimental Group II for fistulotomy and primary reconstruction of anal sphincter The two groups were balanced at a ratio of 1 : 1. The study was carried out under single-blind conditions.

### Inclusion criteria

patients who are above 18 years old and were diagnosed with high trans-sphincteric fistula by Magnetic Resonance Imaging (MRI).

### Exclusion criteria

patient who are less than 18 years old, refusing to do surgery, Inter-sphincteric fistula, horseshoe fistula, Branching fistula, Supra-sphincteric fistula, Previous anal surgery, Inflammatory Bowel Disease as Crohn’s, tuberculosis and patients with history of fecal incontinence.

### Pre-operative

The History of the patients including full personal history, complaint, complete Anorectal examination and assessment of the continence by Wexner score as shown in (Table 1).

**Table 1:** Shows Wexner Incontinence Score

	Never	Rarely	Sometimes	Often	Always
Solid	0	1	2	3	4
Liquid	0	1	2	3	4
Gas	0	1	2	3	4
Wears pad	0	1	2	3	4
Lifestyle Alteration	0	1	2	3	4

Score 0 = perfect 20 = Incontinence. Rarely = Less than once per month, Sometimes = Between once per week and once per month, Often = Between once per day and once per week, Always = At least once per day

### The pre-operative investigations included

- **Laboratory tests**: including routine complete blood count, liver profile, kidney profile, coagulation profile, blood sugar, complete virology screen.
- **Radiological examination**: MRI fistulogram, ECG and echocardiography and stress ECG were performed upon requested by the Anaesthesiologist when indicated.

### Patient counselling and consent

One day before the surgery the patient received a detailed explanation of the types of the surgeries and the expected postoperative complications.

The operative details were explained to help in understanding of the outcome, risks and benefits of the suggested procedure.

An informed consent was taken and signed by the patient and any inquiries, concerns or doubts were discussed with the patient and a first degree relative (upon the patient’s request). The day before surgery, all patients were instructed to have a soft diet and mineral laxative. The night before surgery, all patients had rectal enema with ordinary tap water.

### Operative details

All procedures were performed by the same surgical team under spinal anesthesia in the lithotomy position. All patients had a single dose of 1 g of a third-generation cephalosporin intravenously at the induction of anesthesia.

#### Group (I) underwent modified LIFT

**Fig. 1:**
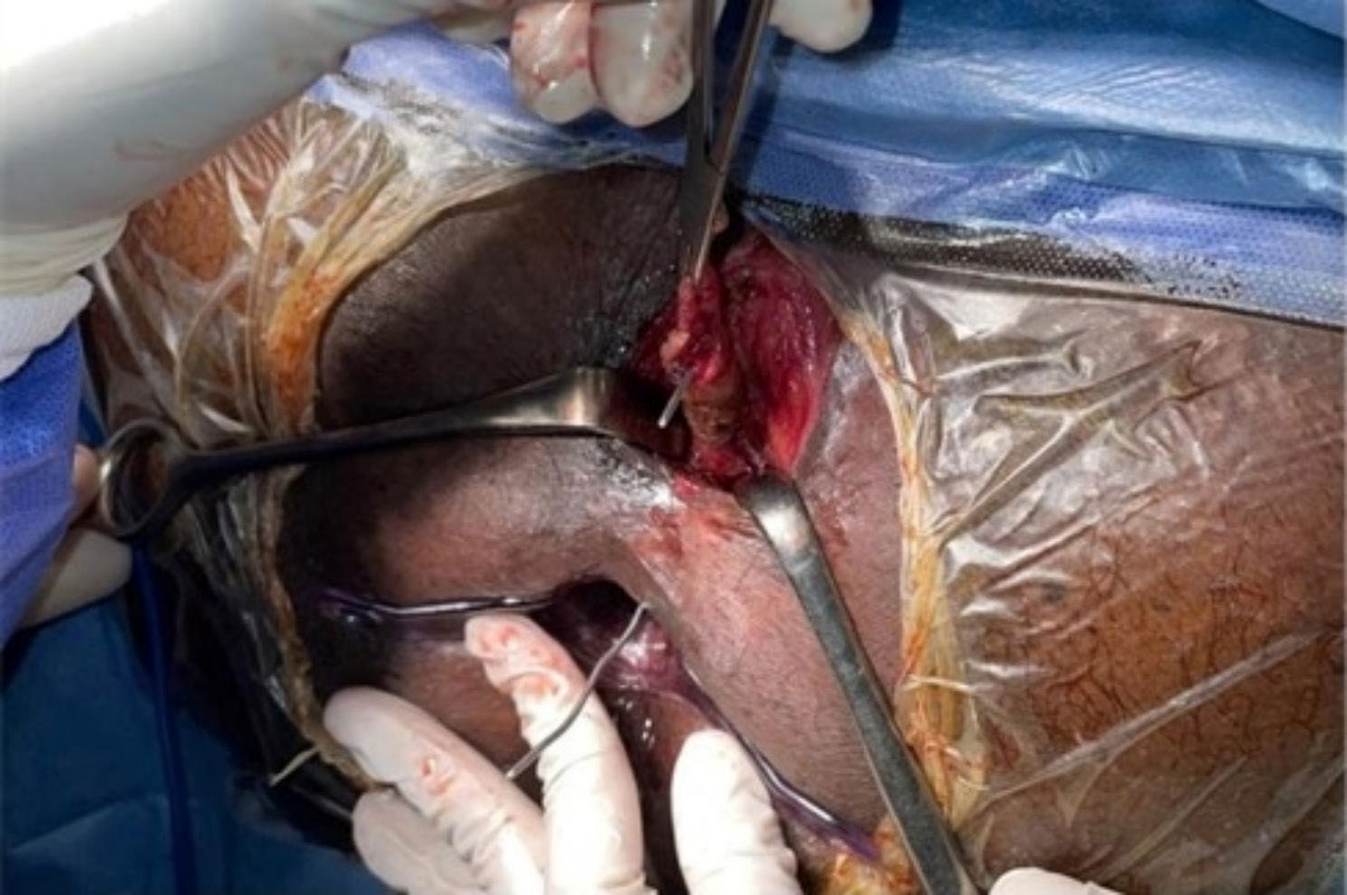
Shows an encircling incision around the external fistula along the fistula tract toward the internal opening

**Fig. 2:**
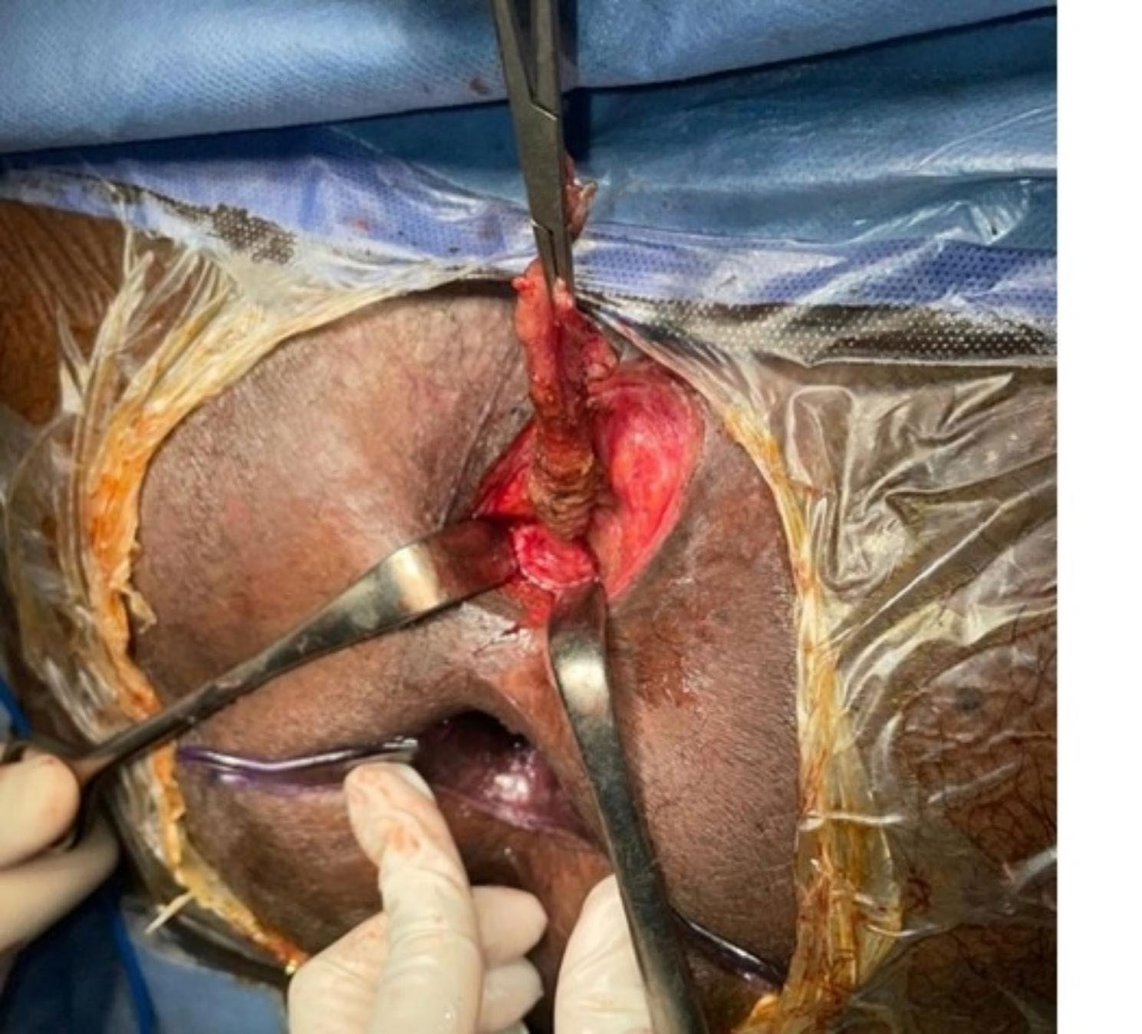
Shows the dissection until the inner layer of the external sphincter separated from the fistula tract

**Fig. 3:**
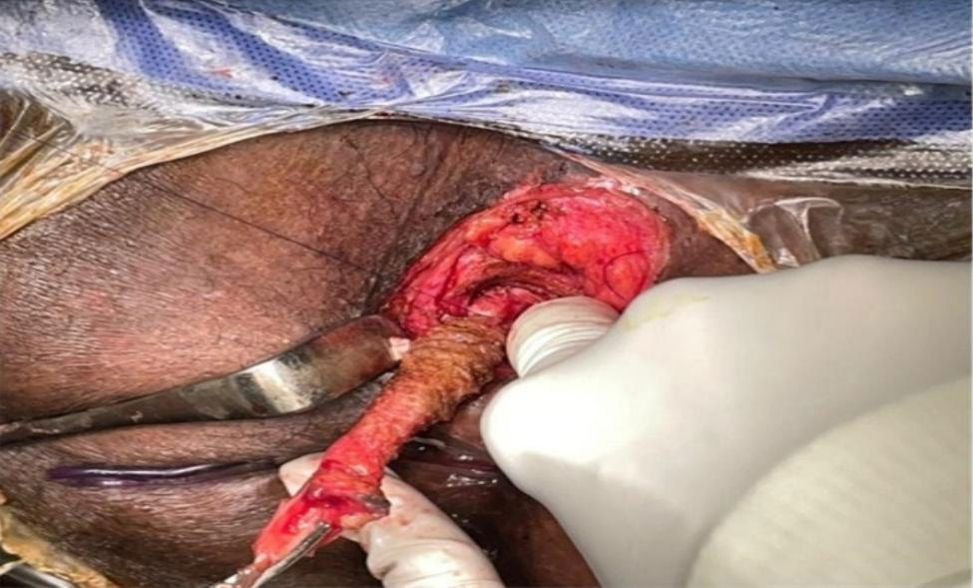
Fistula tract was then ligated with Vicryl 3 − 0 at penetration of the tract in the internal sphincter

**Fig. 4:**
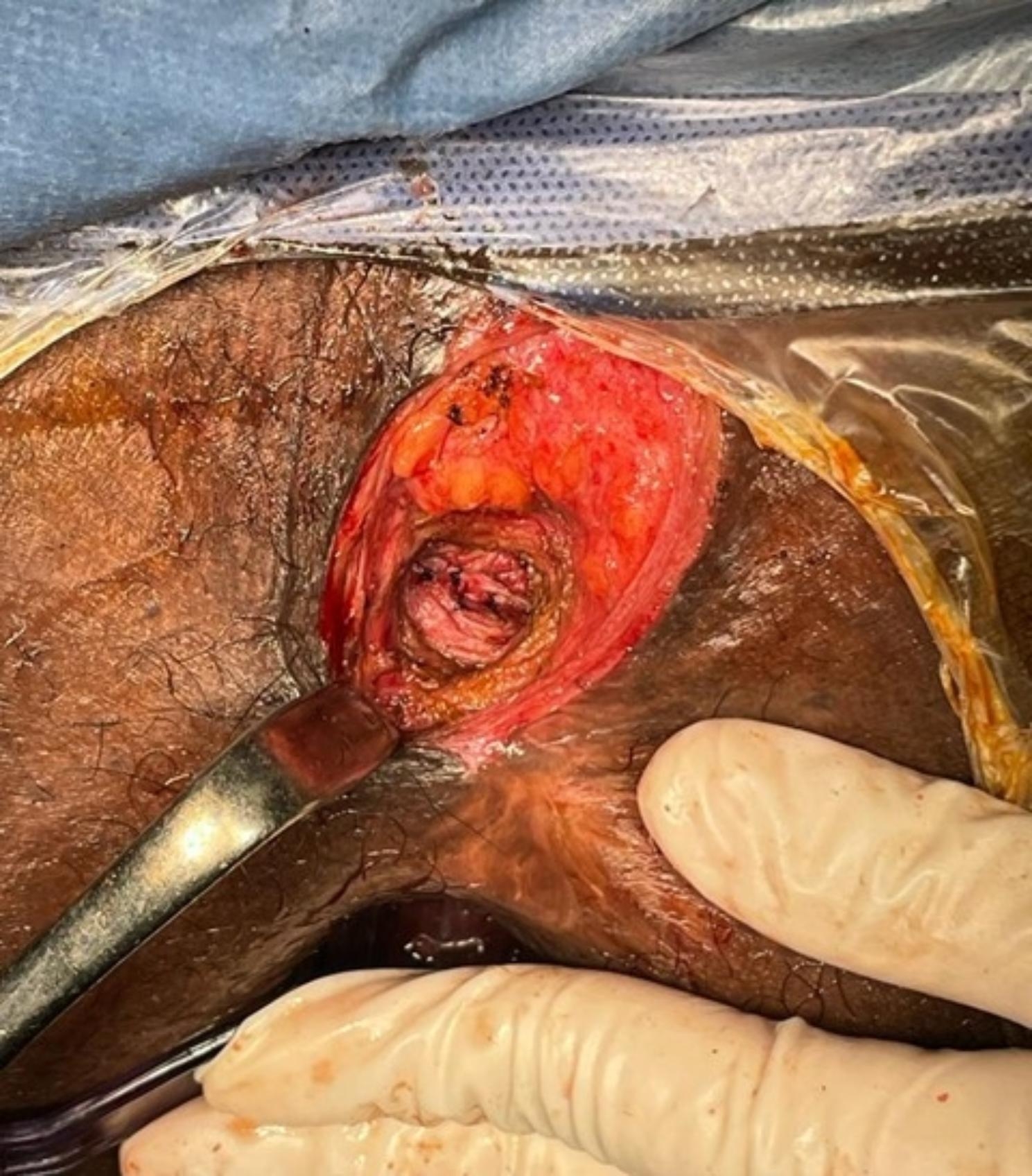
Approximation of the external sphincter edges by PDS 3 − 0

The patient was operated in lithotomy position under spinal anesthesia. The internal opening of the fistula within the anal lumen was visualized using a probe introduced through the external opening. The fistula lumen was rinsed with hydrogen peroxide solution. An encircling incision was made around the external fistula opening, and dissection was started from the external opening, along the fistula tract, toward the internal opening as in Fig. 1. Meticulous dissection was performed around the fistula tract so as not to injure the sphincter muscle, and this was continued until the inner layer of the external sphincter was separated from the fistula tract Fig. 2. The fistula tract was then ligated with Vicryl 3 − 0 at penetration of the tract in the internal sphincter as in Fig. 3. The distal part of was resected using scissors. Another Purse string suture using PDS 4 − 0 was taken at the internal Sphincter edges to bury the proximal stump of the ligated fistula tract. Lastly approximation of the external Sphincter edges using PDS 3 − 0 as in Fig. 4. Hemostasis was achieved using standard unipolar cautery. A cored-out wound was left open for drainage.

#### Group (II) underwent Fistulotomy and primary sphincteroplasty

**Fig. 5:**
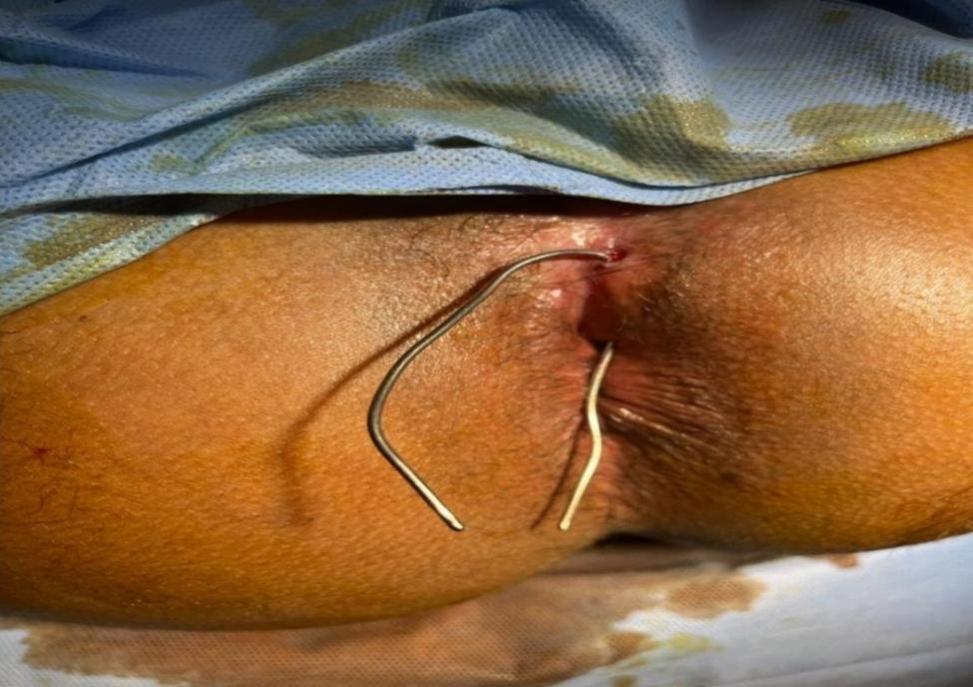
Visualization of the internal opening of the fistula using a probe from the external opening

**Fig. 6:**
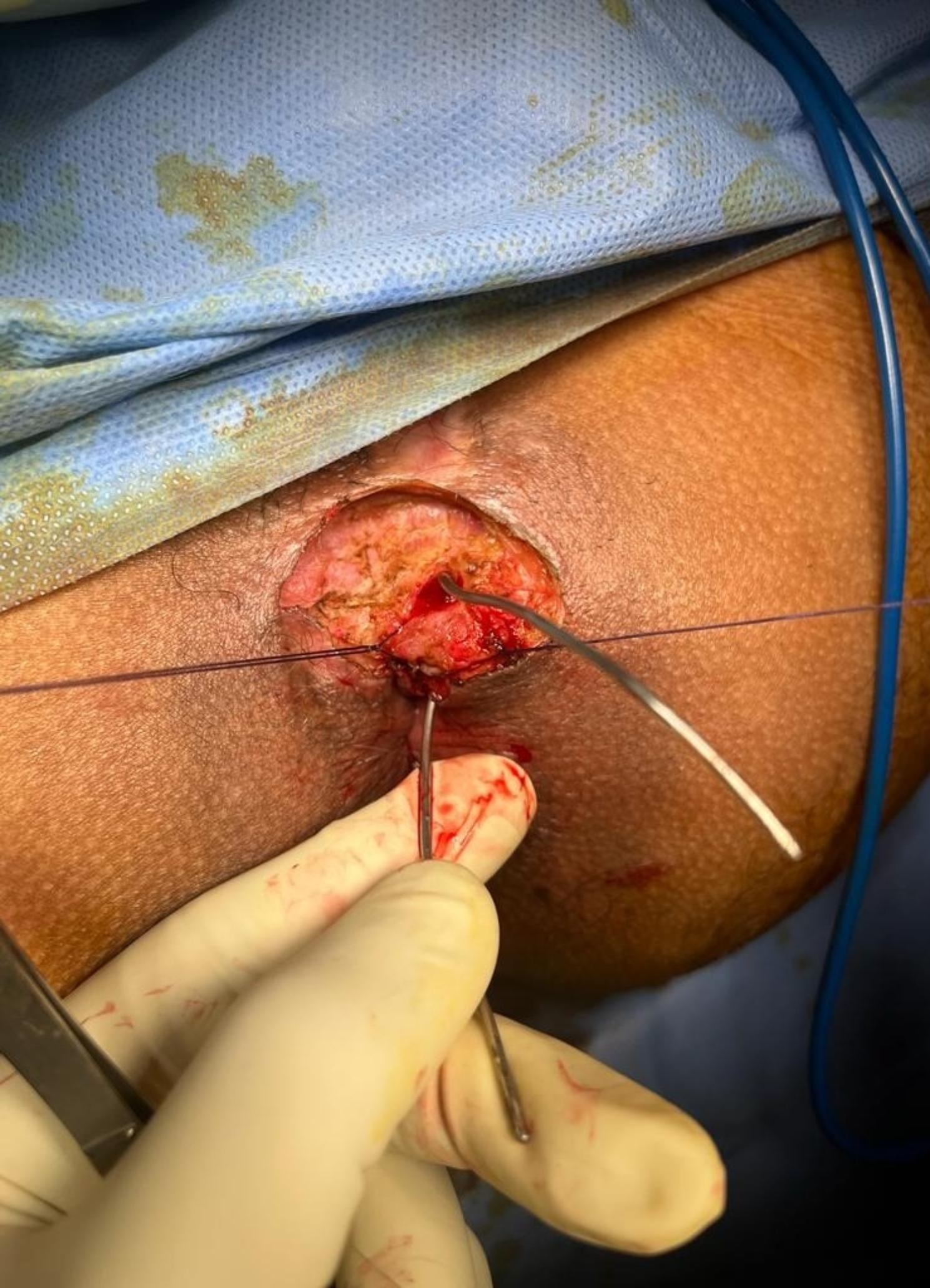
Shows stay sutures through the edges of the sphincter

**Fig. 7:**
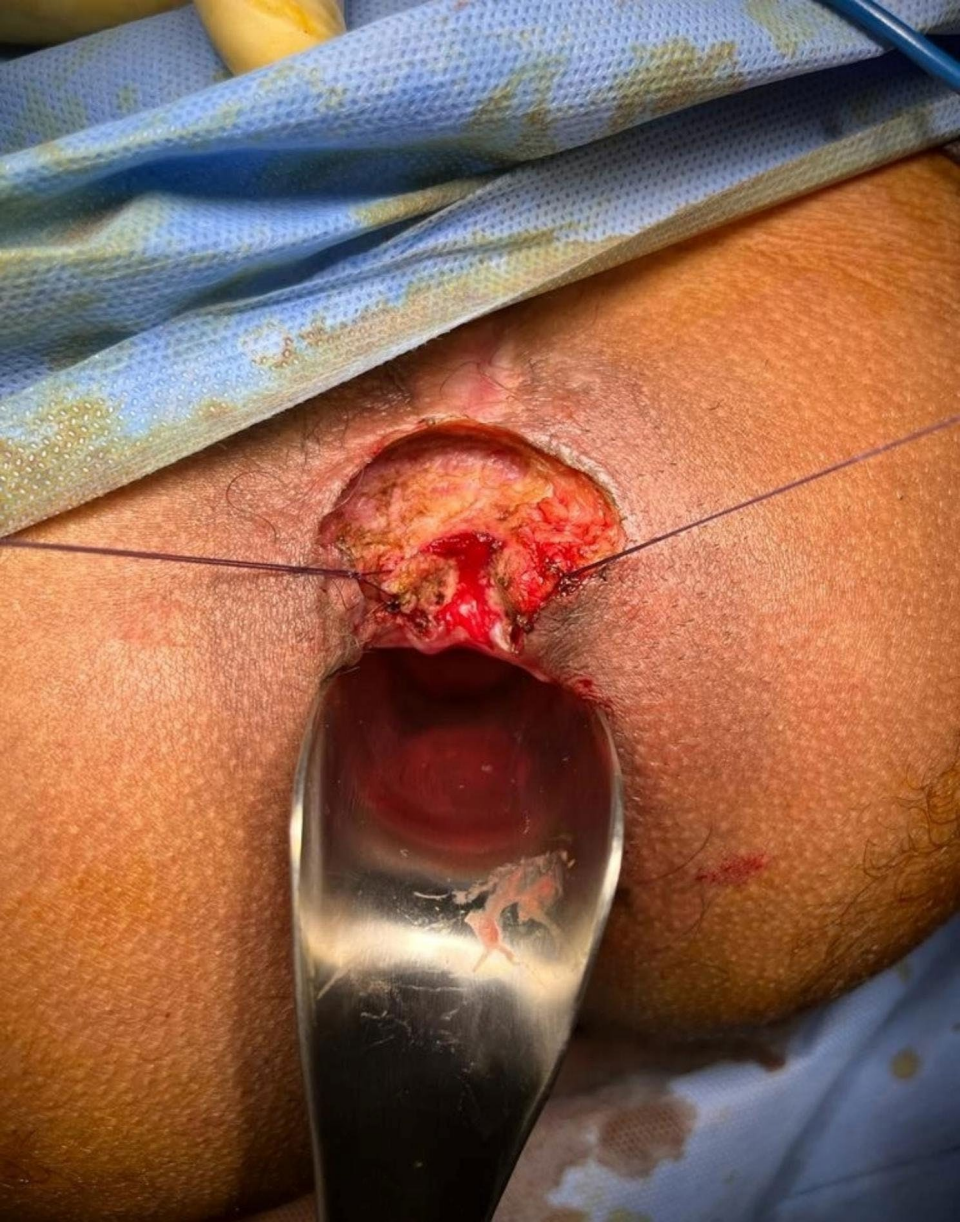
Shows the dissection of the sphincter until the dorsal aspect of the fistula

**Fig. 8:**
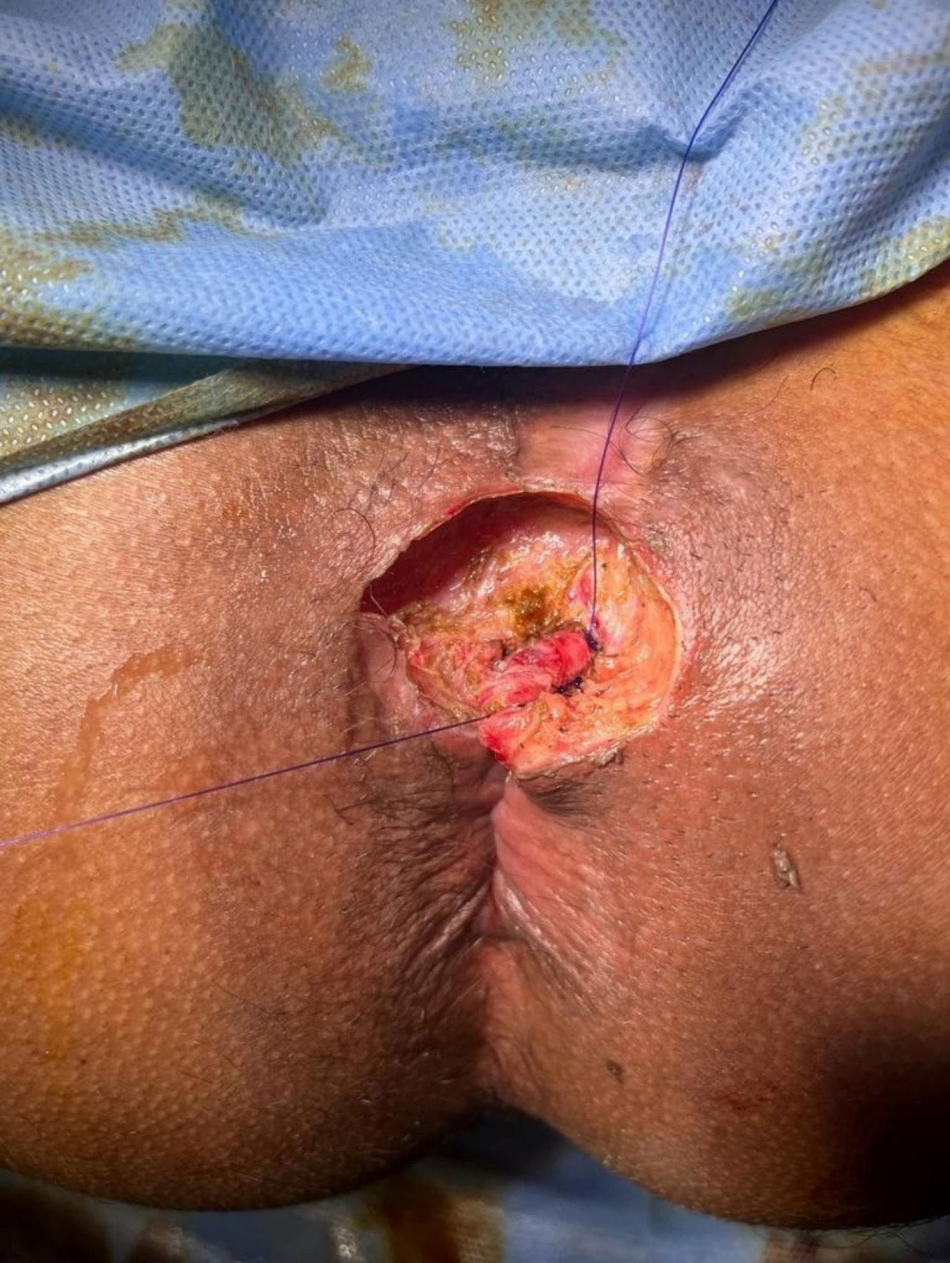
Shows sphincter reconstruction using PDS 3 − 0 transverse mattress sutures

The patient was operated in lithotomy position under spinal anesthesia. The internal opening of the fistula within the anal lumen was visualized using a probe introduced through the external opening as in Fig. 5. Excision of the fistula tract beginning from the external opening toward the lateral edge of the external sphincter and stay suture was taken through edges of the sphincter as in Fig. 6. Dissection of the sphincter until the dorsal aspect of the fistula. Excision and curettage of the granulation as in Fig. 7. The sphincter reconstruction was done using PDS 3 − 0 transverse mattress suture as in Fig. 8. Hemostasis was achieved using standard unipolar cautery. The wound was left open for drainage.

Post-operative work-up and follow-up for one-year duration. Post-operative complications in the form of: wound infection, post-operative pain, early incontinence and recurrence. Patients received oral antibiotics for one week postoperatively. Intake of liquid food was resumed in the evening after the operation they were advised to have a soft diet for 2 days and bulk laxatives for at least 2 weeks. Dressing of the wound was done on the second day postoperatively for all patients. All patients were trained on how to clean themselves and how to do the wound dressing. All Patients were followed up after one week, 2 weeks from discharge then every two weeks until complete healing. Then every two months to complete a one-year follow-up. The fistula was defined to be healed when the external wound healing completed with no discharge. Persistent or recurrent external opening after 2 months of the procedure was considered as recurrence. Patients were observed for the recurrence of the fistula during the follow-up period. None of our patients were lost during the follow-up period.

The initial design of the study included a more follow up time (24 months) but unfortunately, we lost the contact with 6 patients after 12 months follow-up and another 8 patients after 18 months, so we were compelled to reduce our follow-up period to be 12 months.

### Statistical analysis

Data were collected, coded, revised and entered to the Statistical Package for Social Science (IBM SPSS) version 26. The data were presented as number and percentages for the qualitative data, mean, standard deviations and ranges for the quantitative data with parametric distribution. **Chi-square test** was used in the comparison between two groups with qualitative data and **Fisher exact test** was used instead of the Chi-square test when the expected count in any cell found less than 5. The comparison between two independent groups with quantitative data and parametric distribution were done by using **independent t-test**. While the comparison between more than two groups with quantitative data and parametric distribution was done by using **One Way ANOVA**. The confidence interval was set to 95% and the margin of error accepted was set to 5%. So, the *p*-value was considered significant as the following: *P* > 0.05: Nonsignificant (NS). *P* < 0.05: Significant (S). *P* < 0.01: Highly significant (HS).

## Results

Between June 2020 till June 2020 our prospective cohort study was conducted on 80 patients with high trans-sphincteric perianal fistula. From these 80 patients, 55 (68.75%) were males and 25 (31.25%) were females divided into two groups each group consisted of 40 patients. Group (I) subjected to high ligation of the inters-sphincteric fistula tract by lateral approach procedure and Group (II) subjected to Fistulotomy and primary sphincteroplasty. The mean age of group (I) is 46.65 with standard deviation ± 6.6. While, in group (II) the mean age is 45.85 with standard deviation ± 6.07 (*p* = 0.576). As regards co-morbidities of the patients 31(38.75%) were diabetic 14 patients in group (I) and 17 patients in group (II), 19(23.75%) patients were hypertensive 13 in group (I) and 6 in group (II), 11(13.75%) patients were diabetic and hypertensive 8 patients in group (I) and 3 patients in group (II), 19(23.75%) patients had no co-morbidities 5 patients in group (I) and 14 patients in group (II) (p = 0.024).

During the early postoperative period, wound discharge was a constant complaint and presented in all patients of the study (100%). Pain was present in 57(71.25%) patients of the study. As it was present in 25 (62.5%) patients in group(I) and 32 (80%) patients in group (II). Swelling was present in 10 patients of the study; it was present in 4 (10%) patients in group (I) and 6 (15%) patients in group (II). All patients’ characteristics are listed in Table 2.

**Table 2:** shows the patients’ characteristics

Characteristic	Group I	Group II	*P* value
Sex: Male Female	29 11	26 14	0.63^a^
Mean age in years	46.65 ± 6.6	45.85 ± 6.07	0.576^b^
Co-morbidities: Diabetes Hypertension Diabetes and hypertension No co-morbidities.	14 13 8 5	17 6 3 14	0.024^c^

^a^ Fisher exact test

^b^*t*-test

^c^ Chi-square test

The mean time of wound healing was faster in group (I) 4.67 weeks with ± 0.916 standard deviation ranging from 3–7 weeks and the mean in group (II) is 6.05weeks with ± 0.95 standard deviation ranging from 5–8 weeks with high statistical significance as *p*-value is ≤ 0.001. Postoperative wound infection occurred in 2(5%) Patients in group (I) and 7(17.5%) patients in group (II) with no statistical significance (*p* = 0.154). There were no cases of incontinence in group I. However, there were 6(15%) cases of incontinence to gases only scored by Wexner score 3/20 in group II with statistical significance (*p* = 0.026). There was no difference between the two groups in the postoperative pain in which, Postoperative pain was assessed for one week duration by the visual analogue score (VAS) from 0–10 in which, zero is the least and 10 is the maximum. In group (I) 18(45%) patients scored their pain mild from 1–3, 20(50%) patients scored their pain moderate from 4–6 and 2(5%) patients scored severe pain from 7–9 with the mean 1.60 and standard deviation ± 0.59. While, in group (II) 14(35%) patients scored their pain mild from 1–3, 22(55%) patients their pain moderate from 4–6 and 4(10%) patients scored their pain severe from 7–9 with the mean 1.75 and standard deviation ± 0.60 (*p* = 0.275). Recurrence in one-year follow-up occurred in 13(32.5%) patients in group (I), about 7 patients had recurrence in the form of inter-sphincteric fistula and 6 patients in the form of trans-sphincteric fistula. While, in group II recurrence occurred in 1 (2.5%) patient in the form of subcutaneous fistula at the healing site with high statistical significance as *p*-value is ≤ 0.001 as shown in Table 3.

**Table 3:** shows Comparison between the two studied groups according to time of complete wound healing (weeks), Visual Analogue Scale (VAS), incontinence, wound infection and recurrence within one year follow-up

Variables	Group (I) n = 40	Group (II) n = 40	*p*-value
Time of wound healing in weeks Range Mean ± SD	3–7 4.67 ± 0.916	5–8 6.05 ± 0.95	0.001*^, a^
Pain by VAS score Mean ± SD	1.60 ± 0.59	1.75 ± 0.60	0.27^a^
Incontinence	0	6 to gases only	0.026^b^
Recurrence within one-year follow-up	13	1	0.001*^, b^
Wound infection	2	7	0.154^b^

* Significant

^a^*t*-test

^b^ Chi-square test

## Discussion

Anal fistula presents a chronic problem for patients and colorectal surgeons alike. Surgical treatment may result in impairment of continence and long-term risk of recurrence. Treatment options vary according to their location and complexity. The ideal approach should result in low recurrence rates and minimal impact on continence [5].

The present study is a prospective study comparing between ligation of the inter-sphincteric fistula tract by lateral approach (modified LIFT) and Fistulotomy and primary sphincteroplasty in the management of high trans-sphincteric fistulae, first technique was first introduced by Chen et al. as a modification for LIFT technique [2]. The second technique Fistulotomy and primary sphincteroplasty which has a simple idea in the eradicating of infection and restoring the anatomical structure of the sphincter muscles [6]. Although the later technique has sufficiently high success rates, complications such as fecal incontinence and difficult technicality made it not preferred by majority of surgeons [7, 8] .

The mean age of group (I) is 46.65 with a standard deviation of 6.6. While, in group (II) the mean age is 45.85 with standard deviation 6.07 (p = 0.576). From the included 80 patients 55(68.7%) were males and 25 (31.25%) were females (p = 0.469), in addition we found no difference between both groups as regard co-morbidities. These data could eliminate presence of any additional co-factor that could deviate our results as regard to the efficacy and complications of both techniques.

Wound healing was faster in group (I) managed by modified LIFT than in group (II) managed by fistulotomy and primary sphincter reconstruction. The mean time of wound healing in group (I) is 4.67 weeks with ± 0.916 standard deviation ranging from 3 to 7 weeks and the mean in group (II) is 6.05weeks with ± 0.95 standard deviation ranging from 5 to 8 weeks with high statistical significance as *p*-value is ≤ 0.001. this difference sounds logic as we found the size of the exposed wound in group II larger than in group I in addition, there is muscle repair in group II with extension of the wound into the anal canal.

Postoperative wound infection occurred in 2(5%) Patients in group (I) and 7(17.5%) patients in group (II) with no statistical significance (*p* = 0.154). None of our patients in both groups suffered an extensive wound infection needed debridement or any surgical intervention just increasing frequency of dressings and washing of the wound, and we added oral combination of ciprofloxacin 500 mg with metronidazole 500 mg twice daily for only one week and there was no need for any further management except wound care.

As regardpain though the difference between both groups is statistically insignificant(*p* = 0.275) but as 55% of group I patients faced moderate to severe pain (mean 1.60 and standard deviation ± 0.59) in comparison to 65% in group II (mean 1.75 and standard deviation ± 0.60) this could be explained by the increase of the size of wound and inclusion of the anal verge and anoderm in the wound volume in group II patients.

After a one year of follow-up, we noticed a lessrecurrence in group (II) than in group (I). in our opinion this difference sounds logic. The technique of fistulotomy with primary sphincter repair eradicate the pathological tract from the external to the internal opening while with modified LIFT the internal opening still present. Group (I) technique was first introduced by Chen et al. [2], with a recurrence rate 20% as nearly the same recurrence rate revealed by Kang et al. [3], while in our study the modified LIFT technique showed recurrence rate 32%. We had no explanation for the higher rate of recurrence we faced with modified LIFT approach except that we selected a more complex fistula type than the previously mentioned authors. Moreover, others faced a higher recurrence rate asGalan et al. [9] who noticed 37.8% recurrence rate with LIFT approach.

The literature has shown a wide range of recurrence rates following Fistulotomy and primary sphincteroplasty depending on the type and level of complexity of the fistula. According to recurrence rate, Ratto et al. a study included 203 patients with perianal fistula with the recurrence rates 7% in a mean time of follow-up 56 months [10]. In another study, Arroyo et al. showed that the recurrence rate in 70 patients was 8.6% of patients in a follow-up period of 81 months [11]. The rate of recurrence for fistulotomy with primary repair in our study was 2.5%, which is less than other studies and we hypothesize that depend on the shorter follow-up period or the technique of the sphincter reconstruction using a delayed absorbable sutures with proper mobilization of the anal sphincter to avoid tension on the stitches we took for repairing the anal sphincter.

There were no cases ofincontinence in group I as during the procedure, which is a great advantage in this technique, the operator did not cut the anal sphincter but dissected and cored through the external anal sphincter muscles toward the internal opening and stopped any further dissection when exposed the outer fibers of the internal anal sphincter [3].

However, in group II, there were 6(15%) cases of incontinence to gases (Wexner score of 1–3/20) which is a significant difference between the two techniques, A great variability exists within the literature This variability is dependent on the form and degree of complexity of the fistula treatment. Ratto et al. reported That the overall postoperative worsening continence rate was 12.4% (mainly post-defecation soiling) in patients who underwent Fistulotomy or fistulectomy and primary sphincter reconstruction for the treatment of complex anal fistulas [10].

The limitations of this study include the sample size. In addition to, we have nearly no papers comparing both techniques in management of high trans-sphincteric fistula-in-ano. Therefore, further work is still warranted to confirm the long-term outcome of these two techniques.

## Conclusion

Fistulotomy and primary sphincteroplasty is an effective and preferred technique for the trans-sphincteric fistula repair with high statistically significant lower incidence of recurrence in one-year follow-up and better results in affection of the sphincter as compared to modified LIFT technique. In addition, the determination of predictors for failure of both techniques would be useful in defining their roles in the surgical management of all anal fistulas. Moreover, we believe that with proper patients’ selection (without comorbidities that affect healing process negatively) and after failure of sphincter preserving techniques, fistulotomy with primary sphincter repair could be a safe and effective procedure.

## Data Availability

This is a prospective study including 80 patients presented with trans-sphincteric perianal fistula 56 (70%) males and 24 (30%) females divided into two groups each group consisting of 40 patients. Group I subjected to high ligation of the inter-sphincteric fistula tract by lateral approach procedure and Group II subjected to Fistulotomy and primary sphincteroplasty. The study was done from June 2020 to June 2022 including a one-year follow-up postoperative. The datasets used and/or analyzed during the current study available from the corresponding author on reasonable request.

## Declaration

### Declarations

I declare that this manuscript is original, has not been published before, and is not currently being considered for publication elsewhere.

### Clinical Trial details

Trial no.: PACTR202306629134838 Date of Approval:29/06/2023.

## References

[1] Jimenez M, Mandava N. Anorectal Fistula: National Library in Medicine. 2023 Feb 2.

[2] Chen T-A, Liu K-Y, et al. High ligation of the fistula track by lateral approach: a modified sphincter-saving technique for advanced anal fistulas. Colorectal Dis. 2012 Sep;14(9):e627–e30. 10.1111/j.1463-1318.2012.03050.x 22507907

[3] Kang WH, Yang HK et al. High ligation of the anal fistula tract by lateral approach: a prospective cohort study on a modification of the ligation of the intersphincteric fistula tract (LIFT) technique. Int J Surg 2018 Dec.60:9–14. 10.1016/j.ijsu.2018.08.008 30343130

[4] Abbas MA, Jackson CH (2011). Predictors of outcome for anal fistula surgery. Arch Surg.

[5] SCOGLIO D., WALKER A.S. et al. Biomaterials in the treatment of anal fistula: hope or hype, Clin Colon Rectal Surg. 2014;27 (4): 172–81. 10.1055/s-0034-1394156 PMC4226755 25435826

[6] RATTO C., LITTA F. et al. Fistulotomy with endto-end primary sphincteroplasty for anal fistula: results from a prospective study. Dis Colon Rectum. 2013; 56 (2): 226–33. 10.1097/DCR.0b013e31827aab72 23303152

[7] WHITEFORD MH (2005). Standards practice task force; American Society of colon and rectal surgeons. Practice parameters for the treatment of perianal abscess and fistula-in-ano. Dis Colon Rectum.

[8] WEXNER SHAWKIS (2011). Idiopathic fistula-in-ano. World J Gastroenterol.

[9] Placer Galán C (2021). LIFT procedure for posterior fistula-in-ano. Are outcomes good enough? A systematic review and meta-analysisis of observational studies.” “Procedimiento LIFT en fístulas anales de localización posterior. ¿Son buenos los resultados? Revisión sistemática y metaanálisis de estudios observacionales. Cir Esp vol.

[10] RATTO C., LITTA F. et al. Fistulotomy or fistulectomy and primary sphincteroplasty for anal fistula (FIPS): a systematic review. Tech Coloproctol. 2015; 19 (7): 391–400. 10.1007/s10151-015-1323-4 26062740

[11] PÉREZ-LEGAZ ARROYOA (2012). Fistulotomy and sphincter reconstruction in the treatment of complex fistula-in-ano: longterm clinical and manom etric results. Ann Surg.

